# MiR-494-3p promotes PI3K/AKT pathway hyperactivation and human hepatocellular carcinoma progression by targeting PTEN

**DOI:** 10.1038/s41598-018-28519-2

**Published:** 2018-07-11

**Authors:** Hui Lin, Zhi-Ping Huang, Jiao Liu, Yun Qiu, Yuan-ping Tao, Meng-chao Wang, Hui Yao, Ke-zhu Hou, Fang-ming Gu, Xuan-fu Xu

**Affiliations:** 1 The First Department of General Surgeny, Shidong Hospital, Yangpu District, Shanghai, Anhui Medical University, 999 Shiguang Road, Shanghai, 200438 China; 2 Department of Radiotherapy, Shidong Hospital, Yangpu District, Shanghai, Anhui Medical University, 999 Shiguang Road, Shanghai, 200438 China; 3 Department of Gastroenterology, Shidong Hospital, Yangpu District, Shanghai, Anhui Medical University, 999 Shiguang Road, Shanghai, 200438 China; 4 The Third Department of Hepatic Surgery, Eastern Hepatobiliary Surgery Hospital, Second Military Medical University, 225 Changhai Road, Shanghai, 200438 China; 5 Department of Hepatobiliary Surgery, Shanghai Public Health Clinical Center Affiliated to Fudan University, 921 Tongxin Road, Hongkou, Shanghai, 200080 China

## Abstract

Recent studies have shown that miR-494-3p is oncogene and has a central role in many solid tumors; however, the role of miR-494-3p in the progression and prognosis of hepatocellular carcinoma (HCC) remains unknown. In this study, it was found that miR-494-3p was up-regulated in HCC tissues. The high level of miR-494-3p in HCC tumors was correlated with aggressive clinicopathological characteristics and predicted poor prognosis in HCC patients. Functional study demonstrated that miR-494-3p significantly promoted HCC cell metastasis *in vitro* and *vivo*. Since phosphoinositide 3-kinase/protein kinase-B (PI3K/AKT) signaling is a basic oncogenic driver in HCC, a potential role of miR-494-3p was explored as well as its target genes in PI3K/AKT activation. Of all the predicted target genes of miR-494-3p, the tumor-suppressor phosphatase and tensin homolog (PTEN) were identified. In conclusion, the data we collected could define an original mechanism of PI3K/AKT hyperactivation and sketch the regulatory role of miR-494-3p in suppressing the expression of PTEN. Therefore, targeting miR-494-3p could provide an effective therapeutic method for the treatment of the disease.

## Introduction

Hepatocellular carcinoma (HCC), which is the most common liver cancer and the fifth most common cancer, is reckoned as the third leading cause of cancer-related deaths worldwide, because of its poor prognosis due to relapse and metastasis^1–3^. Metastasis remains an essential cause to the high mortality in patients with HCC^4–6^. In order to metastasize, it is required that particular genetic programs should be expressed to activate appropriate interactions with varying microenvironments so as to improve continued survival and proliferation at secondary sites^7–9^. To understand the complex process of metastasis, it is necessary to figure out these genetic programs and the way they affect cellular interactions and signaling cascades^10–12^. Due to the complexity and heterogeneity of HCC, the molecular mechanism underlying its metastasis has not been completely unlocked yet^13–15^.

miRNAs are a group of endogenous, small-size, non-coding RNAs that regulate gene expression through the suppression of translation or the induction of mRNA degradation by hybridizing to the 3′-untranslated region (3′-UTR) of target mRNAs. MicroRNAs (miRNAs) have a central role in a variety of solid tumor processes. MiR-494-3p has been recognized as oncogene in previous studies. Downregulated expression of miR-494-3p could inhibit the invasion and proliferation and promote apoptosis in glioma cells^16^. Overexpression of miR-494-3p in breast cancer stem/progenitor cells could lead to the downregulation of BMI1 protein, the inhibition of the forming capability of mammospheres, and the suppression of their tumorigenicity, and miR-494-3p expression was found to be reversely correlated with patient survival^17^. Moreover, miR-494-3p might suppress prostate cancer progression and metastasis by post-transcriptional regulation to CXCR4 mRNA^18^. However, function and characterization of miR-494-3p in patients with HCC has not been investigated and remains unclear^19–21^.

This study investigated the relationship between levels of miR-494-3p in HCC patient specimens and its outcome and revealed that miR-494-3p could promote the metastasis of HCC *in vitro* and *in vivo*. We also found the molecular mechanism that possibly underlies the functions of miR-494-3p in HCC, which might be that PTEN is a new target gene of miR-494-3p. Taken together, these data indicated that miR-494-3p could be a possible biomarker for diagnosing HCC as well as a target for developing novel therapies for the treatment of HCC.

## Results

### miR-494-3p is up-regulated in HCC tissues and associated with aggressive clinicopathological features

**Figure 1:**
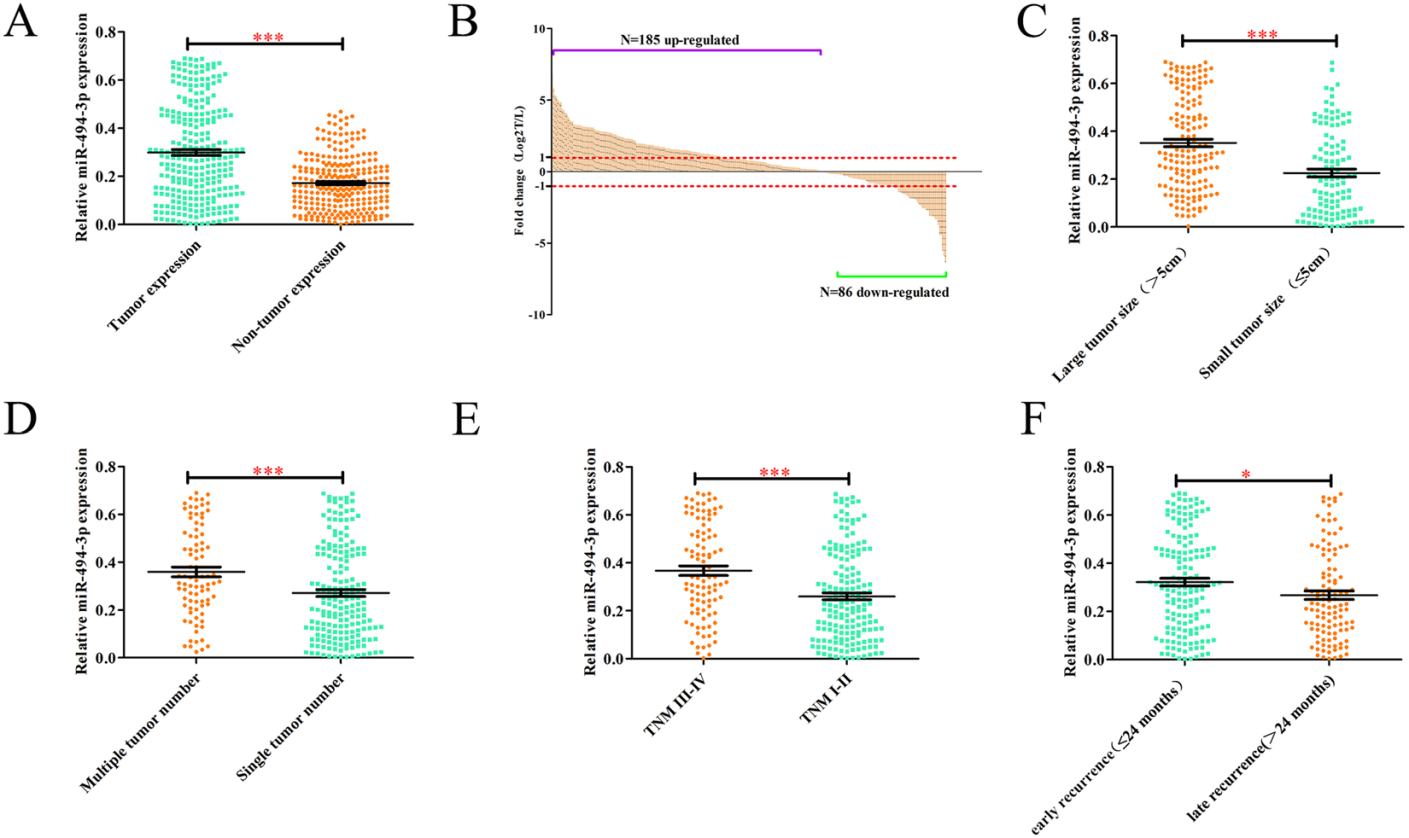
miR-494-3p is up-regulated in HCC tissues and associated with aggressive clinicopathological features. (**A** and **B**) miR-494-3p expression levels were compared between HCC tissue samples and paired adjacent non-tumor tissue samples. U6 was used as an internal control to normalize the expression level of miR-494-3p. (**C**) miR-494-3pexpression levels were examined in HCC tissues with and without vascular invasion. (**D**) miR-494-3p expression levels were examined in larger and smaller HCC tissue samples. (**E**) miR-494-3p levels were compared between TNM III-IV HCC tissues and TNM I-II HCC tissues. (**F**) miR-494-3p levels were compared between HCC tissues exhibiting late recurrence and HCC tissues exhibiting early recurrence. (*p < 0.05, **p < 0.01, ***p < 0.001).

In order to investigate the expression and clinical significance of miR-494-3p in HCC, the expression of miR-494-3p was detected in 271 paired primary HCC tissues and corresponding adjacent non-tumor samples. qRT-PCR indicated that the average expression level of miR-494-3p was significantly higher in the cancerous tissues than in the adjacent non-cancerous tissues (Fig. 1A, p < 0.001). Consistently, miR-494-3p was up-regulated in 68.3% (185/271) of the tested HCC tissues compared to that in matched non-cancerous counterparts (Fig. 1B). Moreover, miR-494-3p expression was increased in HCC patients with larger tumors (>5 cm), multiple tumor number, advanced-stage (stage III-IV) and early recurrence (Fig. 1C–F). To further explore the relations between miR-494-3p expression levels and its clinicopathologic characteristics, we divided the 271 HCC patients into two subgroups, the high and low miR-494-3p expression subgroups, based on the median miR-494-3p expression. As shown in Table 1, high miR-494-3p expression group was correlated with lager tumor size (p = 0.028), larger tumor sizes (≥5 cm) (p = 0.032), multiple tumors (n ≥ 2) (p = 0.041) and advanced TNM stages (p < 0.001). It was indicated by the univariate analysis that of all the clinicopathological characteristics, the miR-494-3p expression level, tumor size, tumor number, vascular invasion, TNM stage, and BCLC stage were correlated with RFS, and the miR-494-3p expression level, tumor size, tumor number, AFP level, vascular invasion, TNM stage, and BCLC stage were correlated with OS (Supplementary Table 1). Moreover, multivariate analysis indicated that miR-494-3p expression levels, along with TNM stage, tumor size and tumor number, are independent risk factors for both recurrence-free survival (RFS) and overall survival (OS) in HCC patients (Table 2). Collectively, these data indicated that increased miR-494-3p expression could be correlated with malignant progression in HCC patients.

**Table 1:** Clinical characteristics of 271 HCC patients according to miR-494-3p expression levels.

Feature	miR-494-3p		χ2	p-value
	Low (n = 135)	High (n = 136)		
**All cases**				
Age, year			0.089	0.807
≥55	61	59		
<55	74	77		
Gender			0.395	0.550
Male	120	124		
Female	15	12		
AFP, μg/L			0.306	0.580
Positive	60	65		
Negative	75	71		
Cirrhosis			0.089	0.802
Present	84	87		
Absent	51	49		
Tumor size,cm			4.605	0.032
≥5	70	88		
<5	65	48		
Tumor number			4.189	0.041
Multiple	35	51		
Single	100	85		
Capsule			1.354	0.273
Present	78	69		
Absent	57	67		
Vascular invasion			2.016	0.171
Present	58	47		
Absent	77	89		
TNM stage			6.777	0.012
III–IV	39	60		
I-II	96	76		
BCLC stage			1.998	0.179
C-D	54	66		
A-B	81	70		

^#^The median expression level was used as the cut-off. Low miR-494-3p expression in each of the 135 patients was defined as a value below the 50th percentile. High miR-494-3p expression in each of the 136 patients was defined as a value above the 50th percentile.

*For analysis of correlation between the expressions levels of miR-494-3p and clinical features, Pearson chi-square tests were used. Results were considered statistically significant at p < 0.05.

**Table 2:** Multivariable analysis of RFS and OS in patients with HCC.

Variable	RFS				OS			
	P	HR	95%	CI	P	HR	95%	CI
Tumor diameter, cm, ≥5 vs. <5	0.000	1.899	1.396	2.583	0.005	1.677	1.173	2.400
Tumor number, multiple vs. solitary	0.001	1.841	1.286	2.635	0.001	2.019	1.354	3.009
Vascular invasion, present vs. absent	0.000	1.940	1.432	2.629	0.000	2.436	1.724	3.443
miR-494-3p, high vs. low	0.035	1.340	1.020	1.759	0.001	1.723	1.252	2.371
TNM stage, III and IV vs. I and II	0.013	1.571	1.098	2.248	0.000	2.209	1.482	3.295
Capsule, absent vs. present	0.707	0.946	0.706	1.266	0.552	0.902	0.644	1.265

### miR-494-3p upregulation predicted poor prognosis in HCC patients

**Figure 2:**
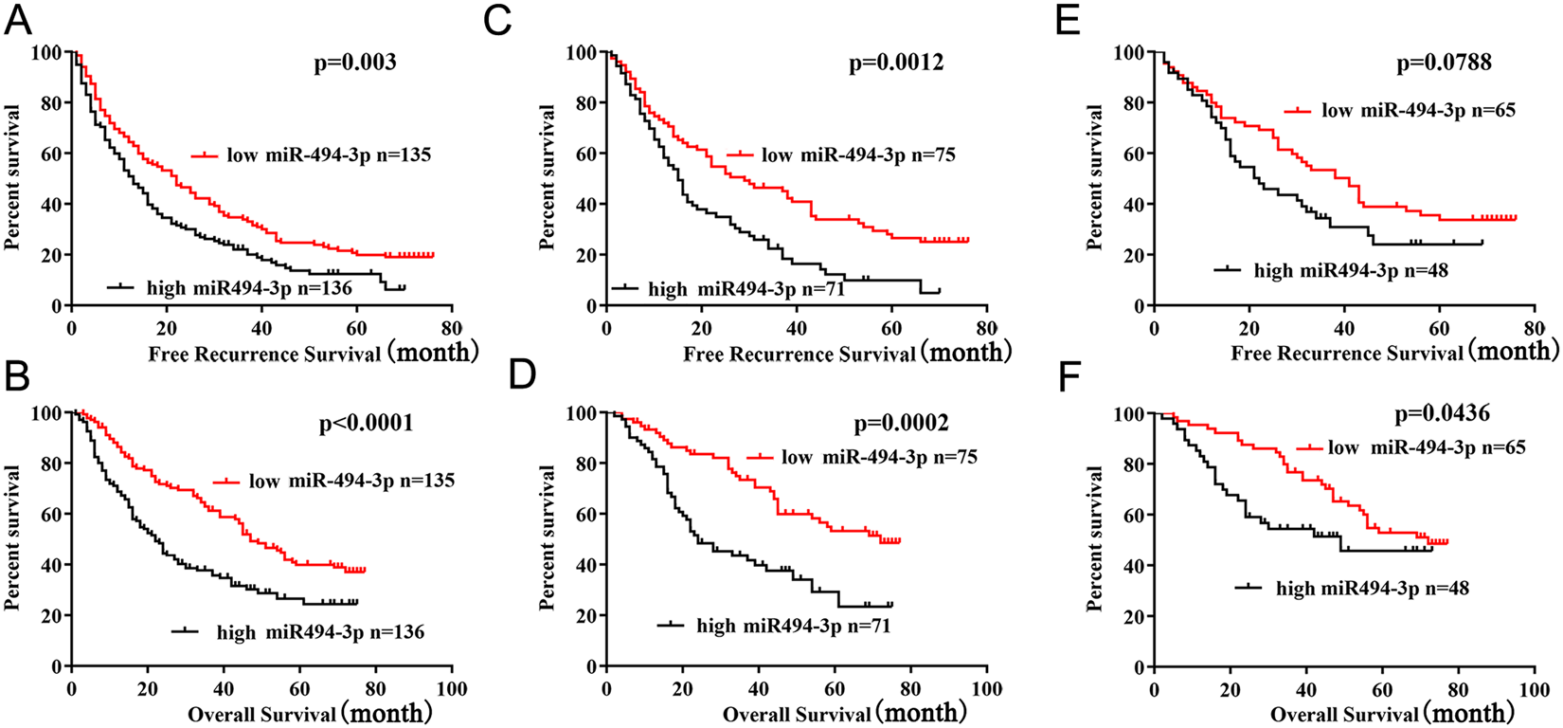
Relationship between miR-494-3p expression and HCC patient prognosis. (**A,B**) The high miR-494-3p subgroup (n = 136) had a significantly shorter RFS and OS than the low miR-494-3p subgroup (n = 135). (**C**,**D**) The prognostic value of miR-494-3p was also observed in patients with a AFP negative: the high miR-494-3p subgroup (n = 71) vs. the low miR-494-3p subgroup (n = 75). (**E**,**F**) The prognostic value of miR-494-3p was also observed in patients with a AFP negative: the high miR-494-3p subgroup (n = 48) vs. the low miR-494-3p subgroup (n = 65). Statistical significance was assessed by two-sided log-rank tests (*p < 0.05, **p < 0.01, and ***p < 0.001).

We further analyzed the association between the miR-494-3p expression and the prognosis of HCC patients after hepatectomy. It was found that the miR-494-3p high-expression group showed significantly poorer RFS (P = 0.003, Fig. 2A) and poorer OS (P < 0.001, Fig. 2B). A Subgroup analysis showed that among patients with AFP negative (146 patients), the difference in RFS and OS between the miR-197-3p high and low-expression groups still existed (P = 0.0012, P = 0.0002; respectively, Fig. 2C,D). Further analysis indicated that of the patients who were tumor size <5 cm (121 patients), the miR-197-3p high-expression group tended to correlate with poor RFS but without statistical significance had poorer (P = 0.0788, Fig. 2E) and poor OS (P = 0.0436; Fig. 2F). As a whole, the data above indicated that the expression level of miR-197-3p could be adopted as an independent factor for predicting the prognosis of HCC.

### miR-494-3p promoted metastasis and invasion of HCC cell *in vitro* and *in vivo*

**Figure 3:**
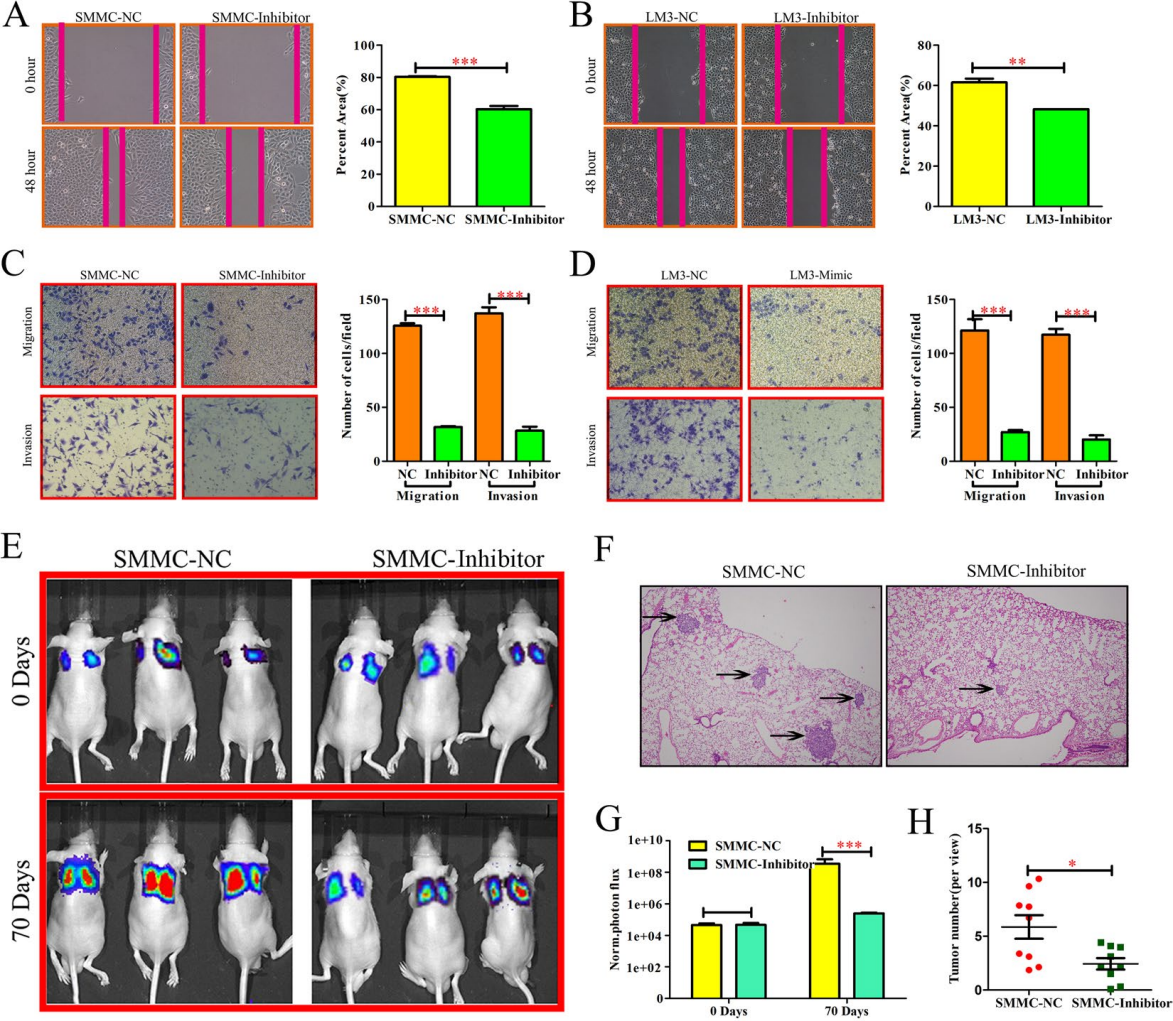
miR-494-3p promoted metastasis and invasion of HCC cell in *vitro* and in *vivo*. (**A,B**) Wound-healing (**C,D**) Transwell migration and Matrigel invasion assays in each HCC cells. Cells were counted in 3 randomized fields at a magnification of 100×. The error bar represents the mean ± SD of triplicate assays. (**E**) Images of lung metastases that developed in the SMMC-7721 cell lines in the lateral tail vein injection models. The images were acquired using an IVIS Imaging System. Representative luciferase signals captured in each group at the time of the initial injection: 0 days and 70 days after cell injection are shown. The statistical analysis is shown in (**F**). (**G**) Representative H&E-stained images of lung metastatic loci from each group in (**A**). The statistical analysis is shown in (**H**) (*p < 0.05, **p < 0.01, and ***p < 0.001).

To explore the basic role of miR-494-3p in HCC progression, firstly we examined the levels of miR-494-3p in several human HCC cell lines (HCCLM3, Hep3B, HepG2, Huh7, and SMMC7721) and normal liver cells (THLE-3). qRT-PCR revealed that miR-494-3p level was markedly increased in all five HCC cell lines compared to that in THLE-3 cell line (Supplementary Fig. 1A). SMMC7721cells and HCCLM3cells were selected for gain- and loss-of-function study. The transfection efficiency was validated by qRT-PCR (p < 0.001, Supplementary Fig. 1B–E). In the wound healing migration assay, microscopic examination at 0 and 48 h showed that SMMC-Inhibitor and LM3-Inhibitor migration were significantly delayed compared with SMMC-NC and LM3-NC migration and invasiveness (P < 0.001; respectively, Fig. 3A,B), however, SMMC-Mimic and LM3-Mimic migration were significantly enhance compared with SMMC-NC and LM3-NC migration (P < 0.001; respectively, Supplementary Fig. 2A,B). Transwell assay also revealed that SMMC-Inhibitor and LM3-Inhibitor cells showed increased migration and invasiveness, compared with other cells (Fig. 3C,D). However, SMMC-Mimic and LM3-Mimic cells showed increased migration and invasiveness, compared with other cells (P < 0.001; respectively, Supplementary Fig. 2C,D). In additional, CCK-8 assay and qRT-PCR indicated that miR-494-3p did not influence HCC cell growth and apoptosis (Supplementary Fig. 3). To verify the function of miR-494-3p *in vivo*, SMMC-NC and SMMC-Inhibitor cells were injected directly into the tail veins of nude mice in order to establish a animal model of lung metastasis. As SMMC-NC and SMMC-Inhibitor express firefly luciferase, the process of lung metastasis for 0 and 70 days was monitored dynamically through an in *vivo* imaging system. The results of the photon flux revealed that miR-494-3p down-expression inhibited lung metastasis (Fig. 3E,F). After 70 days, the lungs were dissected and stained with H&E. It was found that the lungs in the control group displayed markedly more micrometastases, compared with those in the other group (Fig. 3G,H). Collectively, these findings indicated that miR-494-3p could be essential for HCC cell invasive and metastatic potential.

### miR-494-3p represses PTEN expression and activates PI3K in hepatocellular carcinoma cells

**Figure 4:**
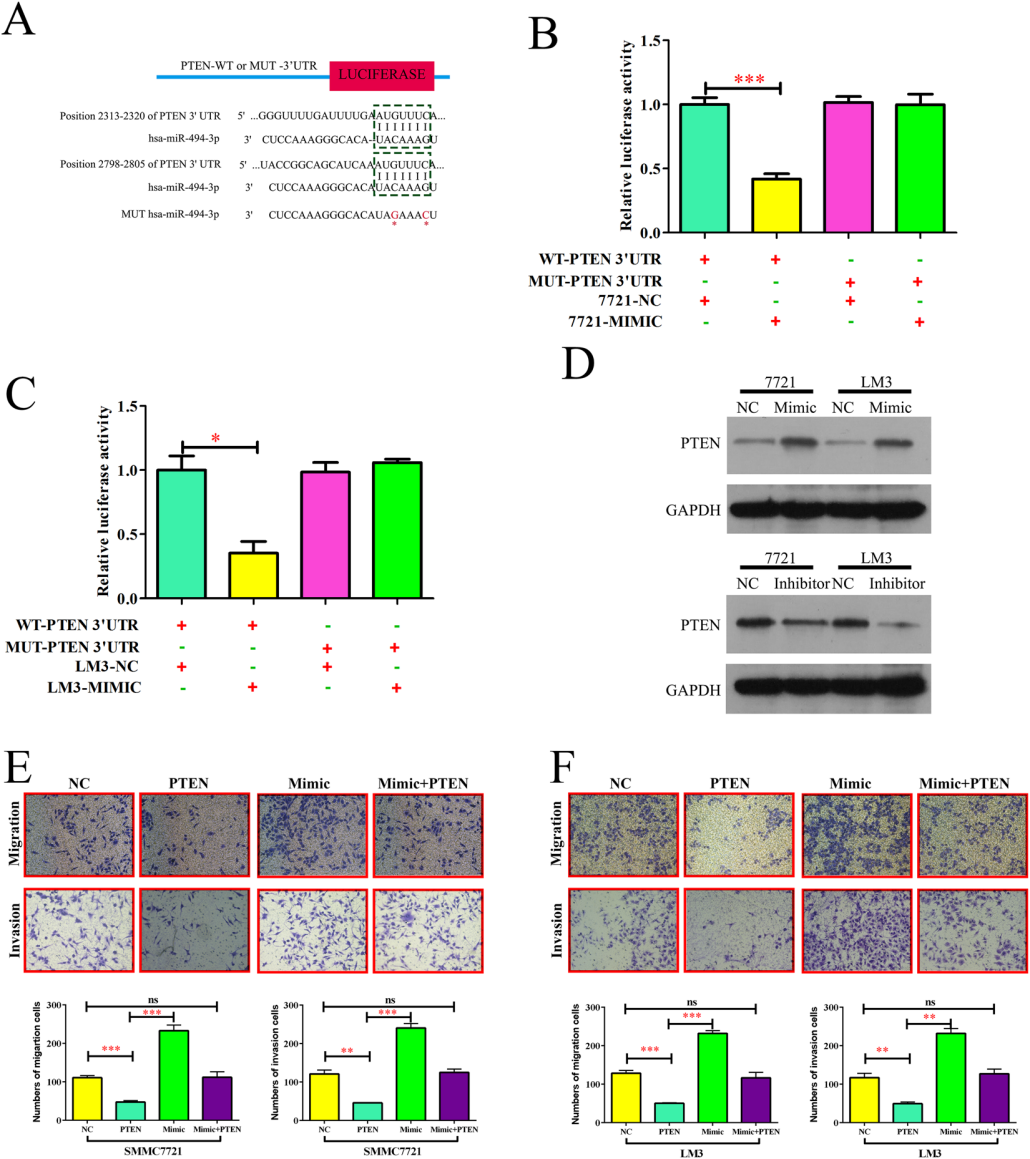
miR-494-3p represses PTEN expression and activates PI3K in human hepatocellular carcinoma cells. (**A**) Sequences showing the putative miR-494-3p binding sites on PTEN 3′UTR and the mutated miR-494-3p binding sites generated by site-directed mutagenesis. (**B**) In SMMC7721 and (**C**) HCCLM3, dual luciferase reporter assay showing significant suppression of luciferase activity only when miR-494-3p was partnered with wild-type PTEN 3′UTR. (**D**) Western blot analysis of PTEN protein expression after transfection in SMMC7721 and HCCLM3 cells. (**E,F**) The migratory properties of the cells were analyzed using the Transwell migration assay with Transwell filter chambers. Results are plotted as the average number of migrated cells from 6 random microscopic fields and the invasive properties of the cells were analyzed with the invasion assay using BioCoat Matrigel invasion chambers. Results are plotted as the average number of invasive cells from 6 random microscopic fields. (*p < 0.05, **p < 0.01, and ***p < 0.001).

To clarify the molecular mechanism underlying the functional effects of miR-494-3p in HCC progression, we looked for candidate target genes of miR-494-3p using in public databases, including TargetScan (http://www.targetscan.org/) and miRanda (microrna.org and miRBase). It was found that the 3′UTR of the tumor-suppressor PTEN mRNA contained the complementary sequence of miR-494-3p (Fig. 4A). To confirm whether miR-494-3p directly targets the 3′UTR of PTEN, we cloned a fragment of the 3′UTR of PTEN mRNA that harbored the predicted binding site of miR-494-3p and inserted it into a luciferase reporter plasmid. Overexpression miR-494-3p significantly suppressed luciferase activity from the wild-type reporter but not from the mutant reporter, which indicated that the 3′-UTR of PTEN could be targeted by miR-494-3p and that the point mutations in this sequence might abolish this effect in SMMC7721 and HCCLM3 (Fig. 4B,C). Besides, the protein levels of PTEN were significantly reduced after overexpression miR-494-3p, while the protein level of PTEN was significantly increased in miR-494-3p knockdown in SMMC-7721 and HCCLM3 cells (Fig. 4D). In contrast, we observed no significant changes for PTEN mRNA levels (Supplementary Fig. 4). These results indicated that miR-494-3p could suppress PTEN protein expression through translational repression.

To determine if the PTEN gene is required for the miR-494-3p’s effects on HCC cell metastasis, ectopic over-expression of PTEN was performed to conduct functional studies in SMMC7721 and HCCLM3 cells. In Fig. 4E,F, the capacities of migration and invasion in PTEN-overexpression HCC cells were significantly inhibited, while overexpression of PTEN abolished the effects of miR-494-3p on of HCC cells migration and invasion ability. Therefore, overexpressed PTEN abolished the effects of miR-494-3p on phenotypes of HCC cells.

By counteracting AKT activation, PTEN serves as a key negative regulator of PI3K signaling. The statuses of the pathway in SMMC-NC and SMMC-Mimic cells were analyzed by measuring the phosphorylation (activation) of AKT. The results revealed that phospho(p) AKT level in SMMC-Mimic cells was markedly higher than that in SMMC-NC cells. Moreover, the level of activated AKT was significantly lowered through the transfection of SMMC-Mimic cells with green fluorescent protein (GFP)-PTEN but not with the empty vector (Supplementary Fig. 5). Taken together, these results could provide a proof of concept for the ability of miR-494-3p to modulate PI3K signaling by silencing PTEN.

## Disscussion

Many oncogenes, growth factors, and tumor suppressor genes have been identified in processes of hepatocarcinogenesis^22–25^, however, the molecular carcinogenic mechanisms and the pathogenic biology of HCC remains unclear^22,26–28^. A growing amount of experimental evidence has been supporting a significant role for miRNAs in tumorigenesis of HCC^27,29,30^.

MiR-494-3p was recognized as an oncogene in lung cancer^31^. Nevertheless, the characterization of miR-494-3p in HCC and its association with cancer progression and development remain unknown. In our study, we found that miR-494-3p was up-regulated in HCC tumor tissues and HCC cell lines. miR-494-3p up-regulation was found to correlated with high, larger tumor sizes (≥5 cm), multiple tumors (n ≥ 2) and TNM stage. The correlation between TNM stage and the expression of miR-494-3p indicates that miR-494-3p may be used as evaluating malignant degree. Furtherly, its correlation with tumor size, tumor number and vascular invasion showed that miR-494-3p played a role in hepatocarcinogenesis.

A subset of patients with tumor size ≤5 cm, who had been predicted to have better outcomes by adopting the standard staging system^32–34^, showed poor prognosis instead, which suggested that a complementary prognostic predictor was needed for the patients. Further prognostic analyses showed that tumor size ≤5 cm with higher miR-494-3p expression also had poorer OS (p = 0.0436). These results indicated that miR-494-3p measurement maybe help clinicians identify the early-stage patients with high recurrence risk and recommend appropriate follow-up and adjuvant therapies for these patients.

Serum AFP, now the most widely-used biomarkers for the diagnosis and treatment of HCC^35–37^, is also used to predict the possibility of recurrence for AFP-positive HCC patients after hepatectomy^38,39^. However, there still lacks a quick, simple and effective marker to monitor the recurrence of the disease and to guide treatments for AFP-negative HCC patients, who account for 30–40% of all HCC patients^40–43^. It was found that in AFP-negative patients, high miR-494-3p expression had significant relationship with poor RFS. MiR-494-3p could also be a potential biomarker for predicting the recurrence risk for AFP-negative HCC patients.

The significant association between miR-494-3p expression in tumors along with the aggressive clinical behaviors and poor prognosis of HCC patients urged us to explore whether miR-494-3p plays a functional role in HCC progression and dissemination. It turned out that both the *in vitro* and *in vivo* data demonstrated that miR-494-3p inhibited hampered the invasion and metastasis of HCC cells; while overexpression miR-494-3p enhanced invasion and metastasis ability of HCC cells. The results also suggested that miR-494-3p could serve as a promising target for therapeutic intervention against invasive and metastatic HCC. Previous reports have shown that the activation of PI3K/AKT contributes to cell growth, promotes invasion and EMT^44–46^. By using TargetScan bioinformatics, this study identified the PTEN gene as a possible direct target for miR-494-3p. Through performing a luciferase reporter assay, real-time PCR and Western blotting, our results verified that PTEN inactivation by miR-494-3p shed light on the mechanism and positive feedback circuits that mediate the activation of the PI3K pathway in HCC carcinogenesis. The role of miR-494-3p/ PI3K/AKT axis in HCC progress might expand the key functional pathways to abnormal invasion of HCC cells.

In summary, we found that miR-494-3p expression was frequently increased in HCC tumor tissues and may serve as a prognostic bio-marker in patients with HCC. Mechanically, our results indicated that miR-494-3p promoted HCC cell metastasis by directly suppressing the expression of PTEN, which not only sheds new light on HCC progression and metastasis, but also provides a potential target for cancer prevention and treatment.

## Materials and Methods

### Ethics statement

All the clinical specimens were approved by the clinical research ethics committee of the Eastern Hepatobiliary Surgery Hospital. Written informed consent was obtained from all patients according to the policies of the committee. Any information that could identify the patients was not included in this article. The animal studies were approved by the Institutional Animal Care and Use Committee of the Second Military Medical University, Shanghai, China.

### Cell culture and transfection

Human hepatocellular cancer cell lines (SMMC-7721, Huh7, HCC-LM3, HepG2, Hep3B and THLE-3) were purchased from the Shanghai Institute of Life Sciences Cell Resource Center in Shanghai, China. All cell lines were cultured in DMEM medium (Hyclone) supplemented with 10% fetal bovine serum (FBS, Life Technologies) and 1% penicillin/streptomycin (Life Technologies, Carlsbad, CA, US). All cell cultures were maintained at 37 °C in a humidified atmosphere with 5% CO_2_. The cells (1 × 10^5^) were seeded into 6-well plates and transfected with either the negative control (NC), miR-494-3p mimic (sense:5′-UGAAACAUACACGGGAAACCUC-3′ antisense: 5′-GGUUUCCCGUGUAUGUUUCAUU-3′), anti-miR-494-3p (5′-GAGGUUUCCCGUGUAUGUUUCA-3′), purchased from GenePharma (Shanghai, China), using Lipofectamine 2000 (Invitrogen) according to the manufacturer’s instructions. Following a 24 h transfection, the media were removed and the cells were placed in complete medium and maintained at 37 °C in an atmosphere of 5% CO_2_. The expression vector pcDNA3.1 containing PTEN was constructed according to the manufacture’s instructions, which was used for “rescue” experiments.

### Patients, tumor tissues and serum samples

A total of 271 pairs of snap-frozen HCC and peritumoral tissues were obtained from the Eastern Hepatobiliary Surgery Hospital. These tissues were used for quantitative real-time polymerase chain reaction (qRT-PCR) analysis. Clinical tissue samples were verified as tumor or non-tumor through ahistopathological examination and the Edmondson grading system. Micrometastases were defined as tumors adjacent to the border of the main tumor as observed by a microscope. Tumor staging was defined according to the sixth edition of the Tumor Node Metastasis (TNM) classification system published by the International Union Against Cancer. The tissue samples were stored at −80 °C until further use. Tumor differentiation was defined according to the R and Barcelona Clinic Liver Cancer (BCLC) staging systems. The study was approved by the Institutional Review Board of the Eastern Hepatobiliary Surgery Hospital. All patients gave their written informed consent to participate in the study. The data do not contain any information that could identify the patients.

### RNA extraction, reverse transcription, and real-time PCR

Total RNA from tissues or cells was extracted through RNA Isolation Kit-miRNeasy Mini Kit (Qiagen, USA) in accordance with the manufacturer’s instructions. Messenger RNA (mRNA) and miRNA were reverse-transcribed of total mRNA through the Revert Aid First Strand cDNA Synthesis Kit (Thermo, USA) in accordance with the manufacturer’s protocol. Complementary DNA (cDNA) was amplified and quantified on CFX96 system (BIO-RAD, USA) using iQ SYBR Green (BIO-RAD, USA). U6 or β-actin was adopted as endogenous controls. Relative fold expressions were calculated by the comparative threshold cycle (2-ddCt) method. Mature miR-494-3p expression was detected using a TaqMan miRNA-assay kit (Applied Biosystems, Foster City, CA, USA) according to the manufacturer’s instructions. RNU6B gene was used as a normalization control. All experiments were performed in triplicate and repeated once. The β-actin gene was used as an internal control. PCR was run using the following conditions: 30 cycles consisting of denaturation at 94 °C for 30 sec, annealing at 56 °C (58 °C for β-actin) for 30 sec, and extension at 72 °C for 30 sec. Each PCR product was separated using 1.5% agarose gel electrophoresis and visualized using ethidium bromide staining. The primer and probe sequences used in the qRT-PCR reactions are listed in Supplementary Table 2.

### Western blotting analysis

Cell protein lysates were separated using 10% sodium dodecyl sulfate polyacrylamide gels, electrophoretically transferred to polyvinylidene difluoride membranes (Roche Diagnostics, Mannheim, Germany), and then detected using PTEN antibodies (ab107918), pAKT (sc-7985-R). Protein loading was measured using a mouse anti-GAPDH monoclonal antibody (mAbcam 8245). Lab Works Image Acquisition and Analysis Software (UVP, Upland, CA, USA) were adopted to quantify the band intensities.

### Luciferase activity assay

The 3′UTR of PTEN was amplified and cloned downstream of the pGL3/Luciferase(Luc)vector. The mutant 3′UTR of PTEN was amplified using the pGL3/Luc-PTEN 3′UTR as the template and then was cloned downstream of the pGL3/Luc vector. For the luciferase reporter assay, cells were co-transfected with either miR-494-3p Inhibitor-s or control and the pGL3/Luc-PTEN 3′UTR or the mutant 3′UTR, together with the controls. At 48 h after transfection, the cells were lysed using RIPA buffer, and luciferase intensity was measured using an F-4500 Fluorescence Spectrophotometer (HIT-ACHI).

### Cell proliferation (MTT) assay and colony formation assay

The transfected cells were plated into 96-well plates at a density of 5,000 cells/well. At 48 h after transfection, the cells were incubated with MTT (3-(4, 5-Dimethylthiazol-2-yl)-2, 5-diphenyltetrazolium bromide) for 4 h at 37 °C. The cells were then agitated with MTT solvent on an orbital shaker for 10 min while avoiding light. The absorbance was measured at 450 nm (OD450nm) using a spectrophotometer.

### Animal studies

To investigate the effects of miR-494-3p on HCC metastasis *in vivo*, the lateral tail vein injection model was adopted to assess the potential of the tumor cells to metastasize to the lungs. The metastases of the lungs were monitored through an IVIS@ Lumina II system (CaliperLife Sciences, Hopkinton, MA, USA) for 10 min after intraperitoneal injection of 4.0 mg of luciferin (Gold Biotech) in 50 µl of saline. The nude mice were housed in cages under standard conditions, and the experiments were performed in accordance with the requirements of the Second Military Medical University Animal Care Facility and the National Institutes of Health guidelines. The mice were maintained in pathogen-free conditions.

### Statistical analysis

All values are presented as means ± standard deviation (SD). Significant differences were determined by GraphPad 5.0 software (USA). Student’s *t*-test was adopted to determine statistical differences between two groups. One-way ANOVA was adopted to determine statistical differences between multiple tests. The chi-square test was used to analyze the relationship between miR-494-3p expression and clinicopathological characteristics. Survival curves were plotted by the Kaplan Meier method and compared by log-rank test. *P* < 0.05 was considered significant. All the experiments were repeated three times.

## Electronic supplementary material

Supplementary materials
