# Supplementary material for: MiR-494-3p promotes PI3K/AKT pathway hyperactivation and human hepatocellular carcinoma progression by targeting PTEN
Source: Sci Rep. 2018 Jul 11;8:10461. doi: 10.1038/s41598-018-28519-2 (PMC6041272; doi:10.1038/s41598-018-28519-2)
Supplement: Supplementary file 1 — Supplementary materials [file 41598_2018_28519_MOESM1_ESM.pdf]

MiR-494-3p promotes PI3K/AKT pathway hyperactivation and human hepatocellular carcinoma progression by targeting PTEN

Hui Lin[1#], Zhi-Ping Huang[4#], Jiao Liu[5#], Yun Qiu[2#], Yuan-ping Tao[4], Meng-chao Wang[4], Hui Yao[2], Ke-zhu Hou[1*], Fang-ming Gu[4*], Xuan-fu Xu[3*]

Supplementary Table 1. Univariable analysis for RFS and OS

Variable	RFS				OS			
	P	HR	95% CI		P	HR	95% CI	
**Age, years,** ≥55 vs. <55	0.700	1.060	0.787	1.428	0.236	1.233	0.872	1.745
**Sex,** male vs. female	0.178	1.378	0.864	2.197	0.471	1.212	0.718	2.045
**AFP, µg/L,** ≥20 vs <20	0.382	1.140	0.850	1.528	0.031	1.451	1.035	2.034
**Cirrhosis** absent vs. present	0.219	0.835	0.626	1.113	0.573	0.907	0.646	1.273
**Tumor diameter, cm,** ≥5 vs. <5	0.001	1.725	1.237	2.406	0.048	1.477	1.003	2.177
**Tumor number,** multiple vs. solitary	0.047	1.501	1.005	2.240	0.047	1.566	1.006	2.439
**Capsule,** absent vs. present	0.752	0.953	0.706	1.286	0.588	0.908	0.641	1.286
**Vascular invasion**, present vs. absent	0.000	1.967	1.439	2.689	0.000	2.458	1.722	3.507
**TNM stage**, III and IV vs. I and II	0.081	1.407	0.958	2.067	0.003	1.886	1.235	2.879
**BCLC stage**, C and D vs. A and B	0.081	1.454	0.955	2.213	0.034	1.651	1.040	2.623
**miR-494-3p expression**, high vs. low	0.027	1.366	1.037	1.801	0.001	1.757	1.272	2.428

Abbreviation: AFP, alpha fetoprotein; BCLC stage, Barcelona Clinic Liver Cancer stage.

Supplementary Table 2 RT-qPCR primers:

Gene	Forward	Reverse
U6	CTCGCTTCGGCAGCACA	AACGCTTCACGAATTTGCGT
PTEN	TGGATTCGACTTAGACTTGACC	AGGATATTGTGCAACTCTGCAA
ACTIN	CACTCTTCCAGCCTTCCTT	CTCGTCATACTCCTGCTTGCT

Supplementary figure 1

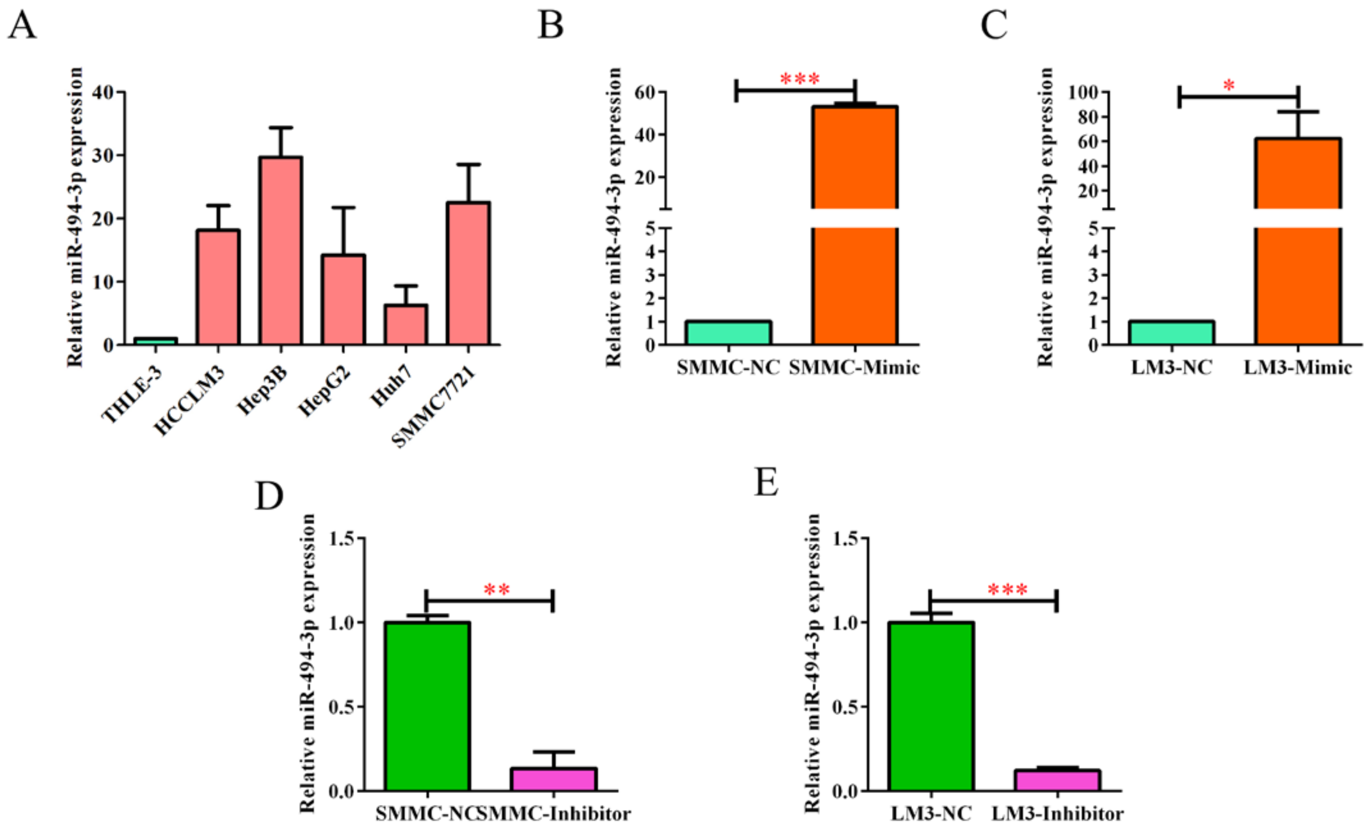

Supplementary figure 2

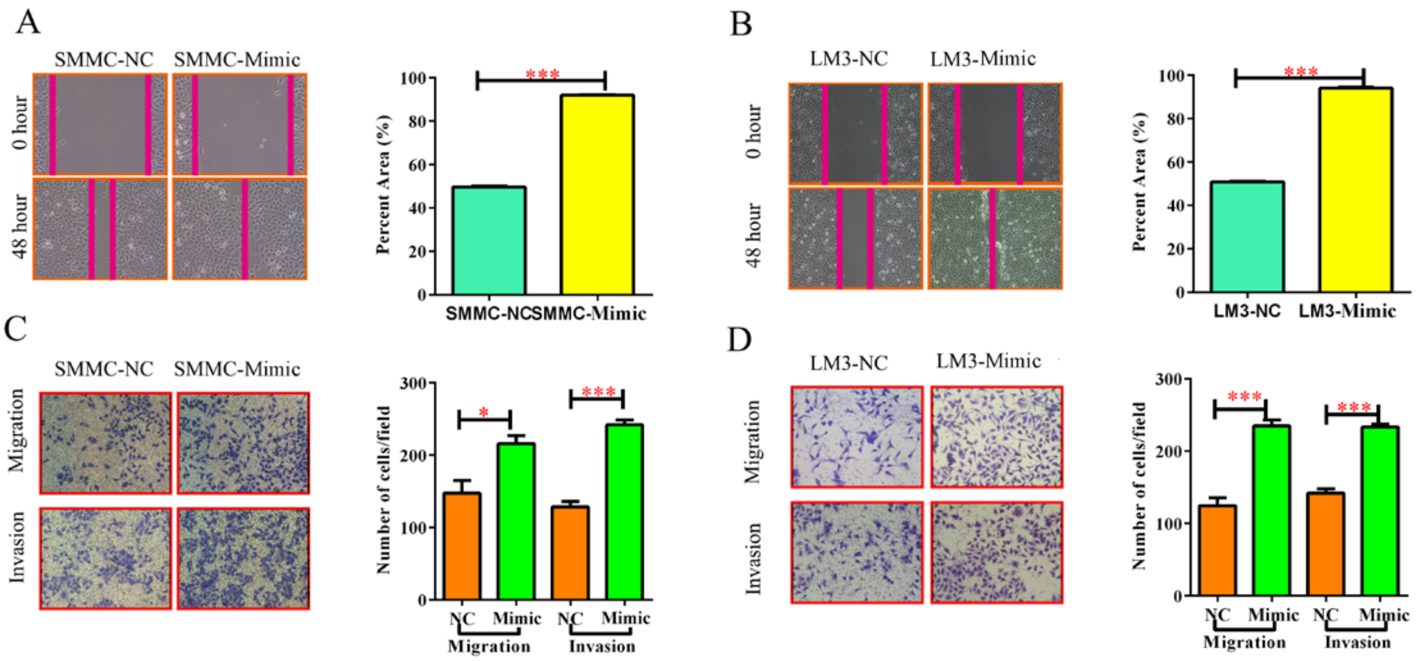

Supplementary figure 3

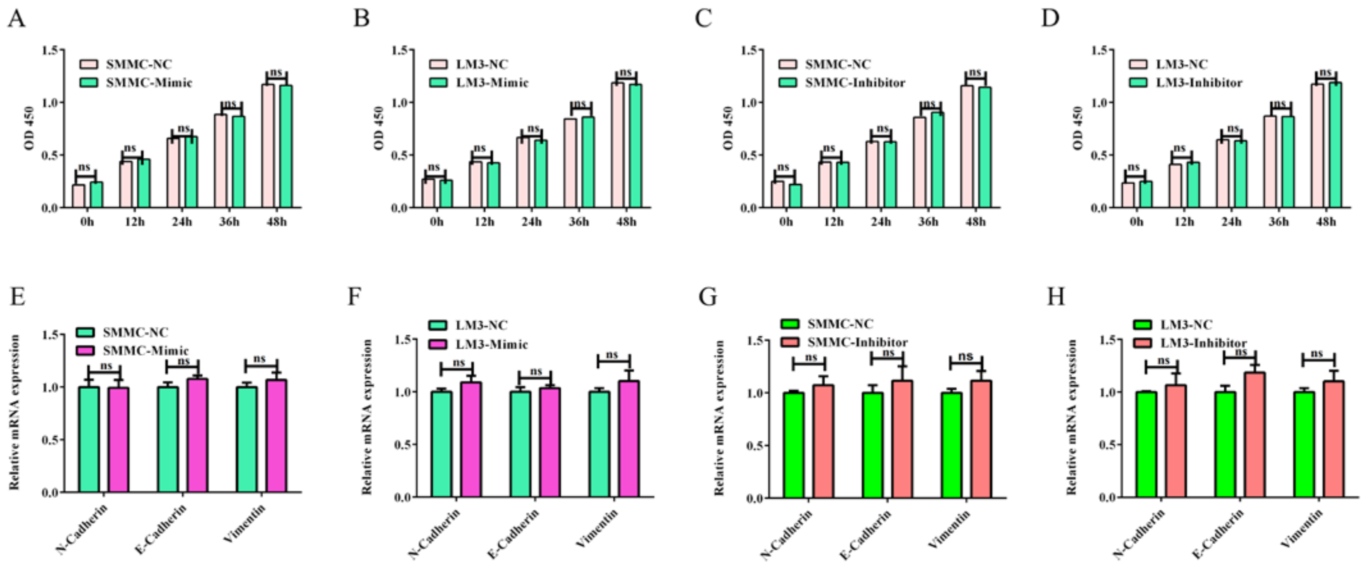

Supplementary figure 4

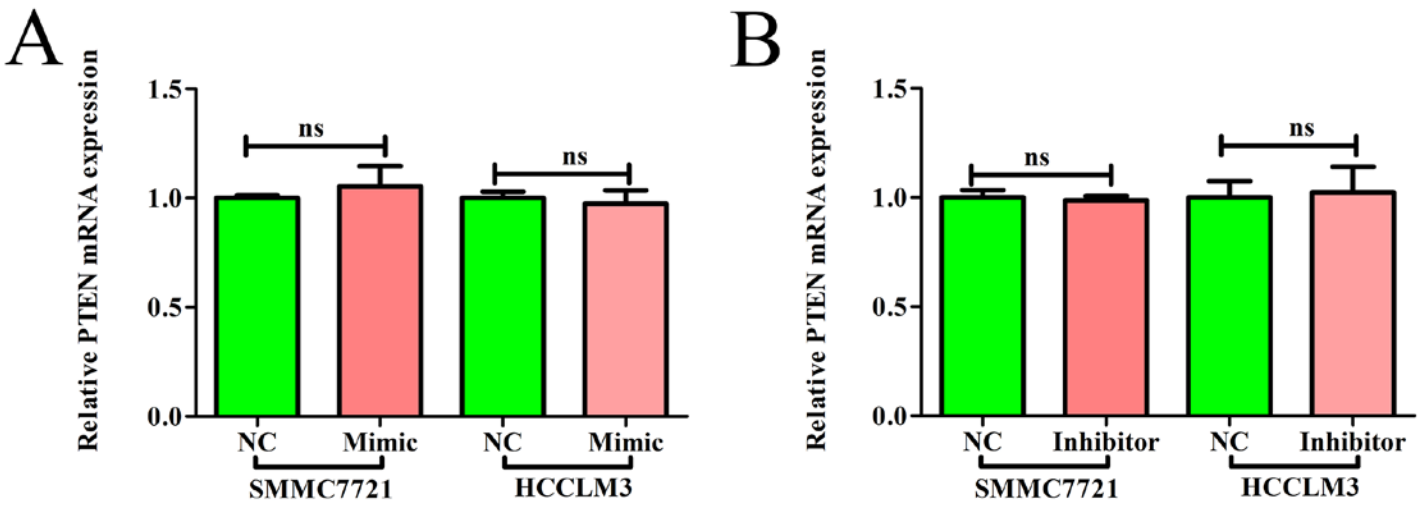

Supplementary figure 5

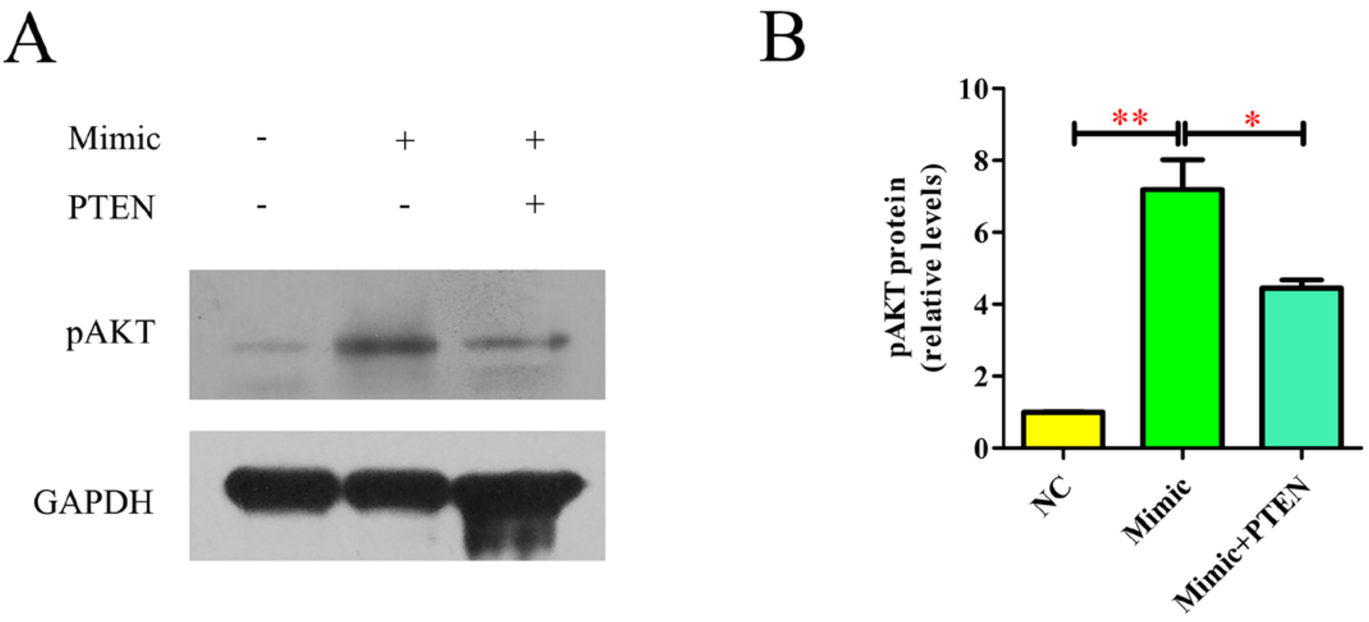

Supplementary legends

Supplement Figure1

(**A**) miR-494-3p expression levels in various human HCC cell lines and human hepatocyte THLE-3 cells. (**B**) miR-494-3p expression levels were significantly increased in SMMC-mimic cells compared with those in SMMC-NC cells. (**C**) miR-494-3p expression levels were significantly inhibited in SMMC-inhibitor cells compared with those in SMMC -NC cells. (**D**) miR-494-3p expression levels were significantly increased in LM3-mimic cells compared with those in LM3-NC cells. (**E**) miR-494-3p expression levels were significantly inhibited in LM3-inhibitor cells compared with those in LM3-NC cells. (*p<0.05, **p<0.01, ***p<0.001)

Supplement Figure2

(**A-B**) Wound-healing (**C-D**) Transwell migration and Matrigel invasion assays in each HCC cells. Cells were counted in 3 randomized fields at a magnification of 100×. The error bar represents the mean±SD of triplicate assays (*p<0.05, ** p<0.01, and *** p<0.001).

Supplement Figure3

(A-D) The growth curves of the SMMC-Mimic, LM3-Mimic, SMMC-Inhibitor, LM3-Inhibitor and their controls, respectively, in CCK8 assay. **(E-H)** Caspase-3, Caspase-7, and Caspase-9 mRNA levels were compared by RT-PCR, (*p<0.05, **p<0.01, ***p<0.001)

Supplement Figure4

(**A**) PTEN mRNA expression in response to miR-494-3p Inhibitor was determined by qRT-PCR in SMMC7721 and HCCLM3. (**B**) PTEN mRNA expression in response to miR-494-3p inhibitor was determined by qRT-PCR in SMMC7721 and HCCLM3 , (*p<0.05, **p<0.01, ***p<0.001)

Supplement Figure5

(**A**) Western blot of pAKT expression (representative of three experiments) 48 h after transfection with GFP-PTEN or empty vector. (**B**) Densitometric quantification. GAPDH was used as a loading control in. Values represent mean ± SD (n = 3). *p < 0.05; **p < 0.01; ***p < 0.001
